# Supplementary material for: Increased risk of adverse events in non-cancer patients with chronic and high-dose opioid use—A health insurance claims analysis
Source: PLoS One. 2020 Sep 14;15(9):e0238285. doi: 10.1371/journal.pone.0238285 (PMC7489518; doi:10.1371/journal.pone.0238285)
Supplement: S1 Table — Adm.R, administration route; O, oral; P, parenteral; R, rectal; SL, sublingual; TD, transdermal; N, nasal; DDD, defined daily dose is the assumed average maintenance dose per day for a drug used for its main indication in adults (29); U, unit; morpheq, Morphine Equivalent Conversion Factor (strength of opioid drug in mg per unit x quantity of units per reimbursed package x number of packages x conversion factor for morphine equivalents. Transmucosal fentanyl conversion MED in milligram for transdermal fentanyl patches were calculated: (mcg/hour (according to the package reimbursed) x 72 hours’ x number of patches per package x number of packages reimbursed x 100 [fentanyl conversion factor]) / 1000. MED in milligram for transdermal buprenorphine patches were calculated: (mcg/h according to the package reimbursed x 96 hours’ x number of patches per package x number of packages reimbursed x 95 [buprenorphine conversion factor]) / 1000. *All DDD are based on the WHO ATC provided daily dose except for codeine. In Switzerland, codeine is available in combination with paracetamol for pain treatment. No DDD from the WHO were available for codeine-combinations. Therefore, the average treatment dose of the combinations was used to calculate DDD: e.g. Co-Dafalgan® four times daily = 4x20mg codeine. (DOCX) [file pone.0238285.s001.docx]

**S1 Table: ATC codes, route of administration and morphine equivalents for opioids**

| **Weak opioids** | | |  |  |  |  |  |  |
| --- | --- | --- | --- | --- | --- | --- | --- | --- |
| **ATC code** | **Adm.R** | | **DDD** | **U** | **Morpheq** | **DDD mg** | **Long / short** | **Substance (example of Brand Names)** |
| N02AA08 | O | | 0.15 | g | 0.15 | 150 | Long | Dihydrocodeine (Treophadol) |
| N02AA59 | O | | 120 | Mg* | 0.15 | 120 | Short | codeine, combinations (Co Bectamol, Co Dafalgan) |
| N02AA59 | R | | 80 | Mg* | 0.3 | 80 | Short | codeine, combinations (Co Bectamol, Co Dafalgan supp) |
| N02AX01 | O | | 0.2 | G | 0.1 | 200 | Short | Tilidine (Valoron capsules or drops) |
| N02AX02 | O | | 0.3 | G | 0.1 | 300 | Short | Tramadol (Tramadol drops) |
| N02AX02 | O | | 0.3 | G | 0.1 | 300 | Long | Tramadol (Tramadol tablets) |
| N02AX02 | P | | 0.3 | G | 0.2 | 300 | Short | Tramadol (Tramadol vials) |
| N02AX02 | R | | 0.3 | G | 0.1 | 300 | Short | Tramadol (Tramadol supp) |
| N02AX06 | O | | 0.4 | G | 0.3 | 400 | Short | Tapentadol |
| N02AX06 | O | | 0.4 | G | 0.3 | 400 | Long | Tapentadol |
| N02AX52 | O | | 150 | mg | 0.1 | 150 | Short | Tramadol combinations |
|  |  | |  |  |  |  |  |  |
| **Strong opioids** | |  |  |  |  |  |  |  |
| **ATC code** | **Adm.R** | | **ddd** | **U** | **morpheq** | **ddd_mg** | **long_short** | **Substance (example of Brand Names)** |
| N02AA01 | O | | 0.1 | g | 1 | 100 | Long | Morphine (MST continus, Morphin HCL) |
| N02AA01 | O | | 0.1 | g | 1 | 100 | Short | morphine (Sevredol Filmtabl., Morphin HCL drops) |
| N02AA01 | P | | 30 | mg | 3 | 30 | Short | morphine (Morphin inj.) |
| N02AA01 | R | | 30 | mg | 1 | 30 | Short | Morphine (Sevredol supp) |
| N02AA03 | O | | 20 | mg | 7.5 | 20 | Long | hydromorphone (Jurnista, Palladon) |
| N02AA03 | O | | 20 | mg | 7.5 | 20 | Short | hydromorphone (Hydromorphon drops) |
| N02AA03 | P | | 4 | mg | 24 | 4 | Short | hydromorphone (Palladon Injection) |
| N02AA04 | O | | 30 | mg | 1 | 30 | Short | nicomorphine (Vilan) |
| N02AA04 | P | | 30 | mg | 1 | 30 | Short | nicomorphine (Vilan vials) |
| N02AA04 | R | | 30 | mg | 1 | 30 | Short | nicomorphine (Vilan supp) |
| N02AA05 | O | | 75 | mg | 1.5 | 75 | Short | oxycodone (Oxynorm kapsules, Oxynorm drops) |
| N02AA05 | O | | 75 | mg | 1.5 | 75 | Long | Oxycodone (Oxycodon or Oxycontin retard) |
| N02AA05 | P | | 30 | mg | 4.5 | 30 | Short | oxycodone |
| N02AA55 | O | | 75 | mg | 1.5 | 75 | Long | oxycodone, combinations (Targin) |
| N02AB02 | P | | 0.4 | G | 0.3 | 400 | Short | Pethidine (Pethidin Vials) |
| N02AB03 | N | | 0.6 | mg | 100 | 0.6 | Short | Fentanyl |
| N02AB03 | SL | | 0.6 | mg | 100 | 0.6 | Short | Fentanyl (Actiq, Abstral, Effentora) |
| N02AB03 | TD | | 1.2 | mg | 100 | 1.2 | Long | Fentanyl (Durogesic, Fentanyl patches) |
| N02AE01 | P | | 1.2 | mg | 90 | 1.2 | Short | Buprenorphine (Temgesic vials) |
| N02AE01 | SL | | 1.2 | mg | 80 | 1.2 | Short | buprenorphine (Temgesic sublingual) |
| N02AE01 | TD | | 1.2 | mg | 95 | 1.2 | Long | buprenorphine (Temgesic patches) |
| N02AF02 | P | | 80 | mg | 1 | 80 | Short | Nalbuphine (Nalbuphin vilas) |
| N07BC01 | SL | | 8 | mg | 60 | 8 | Long | buprenorphine (Subutex) |
| N07BC02 | O | | 25 | mg | 3 | 25 | Long | methadone (Methadon tablets) |
| N07BC02 | P | | 25 | mg | 3 | 25 | Short | methadone (Methadon vials) |
| N07BC02 | R | | 30 | mg | 3 | 30 | Short | methadone (Methadon supp) |

Adm.R, administration route; O, oral; P, parenteral; R, rectal; SL, sublingual; TD, transdermal; N, nasal; DDD, defined daily dose is the assumed average maintenance dose per day for a drug used for its main indication in adults ([29](#_ENREF_29)); U, unit; morpheq, Morphine Equivalent Conversion Factor (strength of opioid drug in mg per unit x quantity of units per reimbursed package x number of packages x conversion factor for morphine equivalents. Transmucosal fentanyl conversion MED in milligram for transdermal fentanyl patches were calculated: (mcg/hour (according to the package reimbursed) x 72 hours’ x number of patches per package x number of packages reimbursed x 100 [fentanyl conversion factor]) / 1000. MED in milligram for transdermal buprenorphine patches were calculated: (mcg/h according to the package reimbursed x 96 hours’ x number of patches per package x number of packages reimbursed x 95 [buprenorphine conversion factor]) / 1000.

*All DDD are based on the WHO ATC provided daily dose except for codeine. In Switzerland, codeine is available in combination with paracetamol for pain treatment. No DDD from the WHO were available for codeine-combinations. Therefore, the average treatment dose of the combinations was used to calculate DDD: e.g. Co-Dafalgan® four times daily = 4x20mg codeine.
