# Supplementary material for: Increased risk of adverse events in non-cancer patients with chronic and high-dose opioid use—A health insurance claims analysis
Source: PLoS One. 2020 Sep 14;15(9):e0238285. doi: 10.1371/journal.pone.0238285 (PMC7489518; doi:10.1371/journal.pone.0238285)
Supplement: S2 Table — (DOCX) [file pone.0238285.s002.docx]

**S2 Table: Summary of codes that define cancer related opioid use**

| **Data source** | **Codes** |
| --- | --- |
| ATC codes for malignant diseases | L01AA, L01AB, L01AC, L01AD, L01AG, L01AX, L01BA (except L01BA01), L01BB, L01BC, L01CA,  L01CB, L01CC, L01CD, L01CX, L01DA, L01DB, L01DC, L01XA, L01XB, L01XC,  L01XD, L01XE, L01XX, L01XY, L03AA, A04AA  L02BA, L02BB, L02BG (except L02BG02), L02BX |
| Tarmed positions | 32.01.01: radiotherapy |
|  | 32.0010: consultation for radiotherapy |
|  | 32.03 – 32.09 Radioonkology specific positions |
|  | 32.0410 1. 32.0440 1  32.04 |
|  | 15.0590 bronchoscopy with removal of tissue endobronchial using an argon beamer  15.06 stent implantation during bronchoscopy |
|  | 19.039 implantation of a stent during a gastroscopy  19.084 implantation of a stent in the pancreas |
