# Supplementary material for: Increased risk of adverse events in non-cancer patients with chronic and high-dose opioid use—A health insurance claims analysis
Source: PLoS One. 2020 Sep 14;15(9):e0238285. doi: 10.1371/journal.pone.0238285 (PMC7489518; doi:10.1371/journal.pone.0238285)
Supplement: S3 Table — (DOCX) [file pone.0238285.s003.docx]

**S3 Table: Definitions of comorbidities**

| **Final category included in the model** | **Original CDS (**[**32**](#_ENREF_32)**)** | **Modified (**[**31**](#_ENREF_31)**)** | **ATC Codes (updated)** |
| --- | --- | --- | --- |
| Chronic Infections | HIV | HIV | J05AE, J05AF, J05AG, J05AX07, J05AX08, J05AX09, J05AR (except J05AF05, J05AF08, J05AF10, J05AF11, J05AF12) |
|  |  | Hepatitis (viral) Viral Hepatitis | J05AB04 (Ribavirin), J05AF05 (Lamivudine), J05AF08 (Adefovir), J05AF10 (Entecavir), J05AF11 (Telbivudine), L03AB04 (interferon alpha), L03AB05, L03AB10, J05AX15 (Sofosbuvir), J05AX16 (Dasabuvir) |
|  | Tuberculosis | Tuberculosis | 1 of the following: J04AM02, J04AM03, J04AM05, J04AM06 (combinations of drugs)  OR  Combination of 3 of the following codes: J04AB02 (Rifampicin), J04AC01 (INH), J04AC51 (INH combinations), J04AK01 (Pyrazinamide), J04AK02 (Ethambutol) |
|  | Chronic osteomyelitis and complicated nosocomial infections | Osteomyelitis and complicated nosocomial infections | 2 drug combination: i.e. Rifampicin + 1 other ATC code  J04AB02 Rifampicin + J01MA Fluoroquinolones (Levofloxacin, Ciprofloxacin) OR  J04AB02 Rifampicin + Clindamycin (several codes: D10AF01 / G01AA10/ J01FF01 / D10AF51 OR  J04AB02 Rifampicin + J01XX09 daptomycin OR  J04AB02 Rifampicin + J01XX08 linezolid |
|  | Nosocomial skin infection | Nosocomial infections | J01XX08 (Linezolid), J01XX09 (Daptomyzin) |
| Inflammatory diseases (autoimmune rheumatoid, gastrointestinal) | Inflammatory diseases including rheumatic conditions, crohn’s disease, ulcerative colitis, autoimmune diseases |  | H02A, H02B, L04AB01, L04AB04, A07EC, M01CB, P01BA02, and L01BA01 with maximal dose 30mg/day and no malignancy defining codes within the 3 months rule |
| Renal disease | Renal disease |  | V03AE (drugs for hypercalcemia and hyperphosphatemia), A11CC03 (active vitamin d), A11CC04, A11CC07 |
| End stage renal disease | End stage renal disease  (ESRD) |  | B03XA (Epo) |
| Diabetes | Diabetes |  | A10AB, A10AC, A10AD, A10AE, A10AF, A10BA, A10BB, A10BC, A10BD, A10BF, A10BG, A10BH, A10BX |
| Pulmonary disease | Cystic fibrosis | Cystic fibrosis | R07AX02 |
|  | Respiratory illness, asthma |  | R03AC, R03AH, R03AK, R03BA, R03BB, R03BC, R03BX, R03CC, R03CK, R03DA,  R03DB, R03DC, R03DX |
| Liver failure |  |  | A06AD11, H01CB01, H01CB02, H01BA01, H01BA04 |
| Organ transplant |  |  | L04AA, L04AC01, L04AC02, L04AD01, L04AD02 |
| Neurological diseases | Amyotrophic lateral sclerosis (ALS) |  | N07XX02 Riluzone (Rilutec®) |
|  | Multiple sclerosis (MS) |  | L04AA23 natalizumab, L04AA27 fingolimod |
|  | Parkinson’s disease |  | N04BA, N04BB, N04BC, N04BD, N04BX (**except** N04BC01, N04BC06, N04BC07,  N04BC08) |
| Cardiovascular disease | Coronary and peripheral vascular disease | Antiplatelet | B01AC, C04AD03 (except B01AC06, B01AC08, B01AC09, B01AC11, B01AC15,  B01AC19, B01AC21) |
|  |  | Anticoagulant | B01AA (vitamin k antagonists), B01AB (heparin), B01AD (streptokinase = enzymes), B01AE (thrombin inhibitors), B01AF (factor Xa inhibitors), B01AX |
|  | Cardiac disease  ASCVD | Anti-arrhythmic | C01AA (digitalis), C01BA (antiarrhythmics class Ia), C01BB, C01BC, C01BD (amiodarone), C01BG, C01EB10 (Adenosin -> AV-reentry tachycardia) |
|  |  | Ischemic heart disease | C01DA (nitrates), C01DX16 (vasodilators) |
|  | Hypertension |  | C02AA02 (central antiadrenergic agents), C02AB02, C02AC, C02BA, C02BB, C02CA, C02CC, C02DA, C02DB,  C02DD, C02DG, C03AA, C03AB (Thiazides and potassium), C03AX, C03DA, C03DB, C02L |
|  |  | Ischemic heart  disease /  Hypertension | C07AA (non-selective BB), C07AB (selective BB), C07AG (alpha- and beta blockers), C07BA (BB + Thiazides), C07BB, C07BG, C07CA, C07CB, C07CG, C07DA,  C07DB, C07EA, C07EB, C07FA, C07FB, C08CA (Calcium channal blockers), C08CX, C08DA, C08DB, C08EA,  C08EX, C08GA |
|  | Congestive heart  Failure | Congestive heart  failure / Hypertension | C01CA07 (doputamine), C01CE01 (amrinone), C01CE02, C01EB09, C03CA (diuretics), C03CB, C03CC, C09AA (ACE nhibitors), C09BA,  C09BB, C09CA, C09DA, C09DB, C09DX04 (valsartan and sacubitril) |
| Thyroid disorders | Thyroid disorders |  | H03AA, H03BA, H03BB |
| Gout | Gout |  | M04AA, M04AB, M04AC01 |
| Psychiatric disease | Depression |  | N06AA, N06AB, N06AF, N06AG, N06AX (except N06AX01, N06AX02) |
|  | Psychotic illness |  | N05AA, N05AB, N05AC, N05AD, N05AE, N05AF, N05AG, N05AH, N05AL,  N05AX (except N05AD08, N05AL01) |
|  | Bipolar disorders |  | N05AN |
