# Supplementary material for: Increased risk of adverse events in non-cancer patients with chronic and high-dose opioid use—A health insurance claims analysis
Source: PLoS One. 2020 Sep 14;15(9):e0238285. doi: 10.1371/journal.pone.0238285 (PMC7489518; doi:10.1371/journal.pone.0238285)
Supplement: S4 Table — (DOCX) [file pone.0238285.s004.docx]

**S4 Table: Full models to predict adverse events**

|  | **ED visits** | **Infections** | **Hospitalization** | **Death** |
| --- | --- | --- | --- | --- |
|  | OR (95% CI) | | | |
| Age | **1.011 (1.010; 1.012)** | **0.996 (0.995; 0.997)** | 0.999 (0.998; 1.000) | **1.086 (1.084; 1.089)** |
| Female | 0.971 (0.933; 1.010) | **1.183 (1.139; 1.228)** | **0.949 (0.912; 0.987)** | **0.598 (0.565; 0.633)** |
| (Semi)private insurance | 0.989 (0.945; 1.034) | 1.020 (0.976; 1.065) | **1.127 (1.077; 1.178)** | **0.888 (0.834; 0.946)** |
| Managed care | 1.012 (0.967; 1.060) | 0.963 (0.923; 1.005) | **1.530 (1.464; 1.599)** | **0.814 (0.759; 0.872)** |
| Italian / French part | **0.844 (0.804; 0.885)** | **1.235 (1.181; 1.292)** | **0.935 (0.891; 0.980)** | **0.804 (0.751; 0.861)** |
| Daily dose (reference <20 mg) |  |  |  |  |
| 20 - <50 mg | 1.009 (0.939; 1.084) | **0.901 (0.845; 0.960)** | **1.105 (1.030; 1.186)** | 1.037 (0.925; 1.163) |
| 50 - <100mg | 1.046 (0.976; 1.121) | **0.927 (0.871; 0.987)** | **1.175 (1.098; 1.258)** | **1.191 (1.070; 1.327)** |
| ≥100 mg | **1.207 (1.129; 1.289)** | 0.993 (0.935; 1.055) | **1.289 (1.207; 1.377)** | **1.665 (1.502; 1.847)** |
| Duration (reference acute) |  |  |  |  |
| subacute | 0.967 (0.892; 1.049) | **1.256 (1.171; 1.346)** | **1.192 (1.103; 1.288)** | **0.734 (0.656; 0.822)** |
| chronic | **1.089 (1.034; 1.146)** | **1.737 (1.660; 1.817)** | **1.223 (1.162; 1.286)** | **0.613 (0.571; 0.659)** |
| very chronic | **1.758 (1.666; 1.855)** | **4.154 (3.945; 4.373)** | **1.824 (1.728; 1.925)** | **0.464 (0.428; 0.502)** |
| Benzodiazepine | **1.464 (1.407; 1.523)** | **1.183 (1.138; 1.230)** | **1.118 (1.073; 1.164)** | **1.450 (1.371; 1.535)** |
| Pharmacologic substance |  |  |  |  |
| Morphine | **1.828 (1.751; 1.907)** | **1.296 (1.240; 1.354)** | **1.278 (1.223; 1.336)** | **5.051 (4.770; 5.349)** |
| Oxycodone | **1.161 (1.110; 1.215)** | **1.146 (1.094; 1.200)** | **2.715 (2.595; 2.840)** | **0.777 (0.728; 0.829)** |
| Fentanyl | **1.177 (1.124; 1.233)** | **1.178 (1.123; 1.235)** | **1.181 (1.127; 1.238)** | **2.034 (1.912; 2.164)** |
| Pethidine | **1.406 (1.312; 1.507)** | **1.714 (1.603; 1.833)** | **1.178 (1.097; 1.265)** | **0.698 (0.604; 0.805)** |
| Buprenorphine | **1.148 (1.075; 1.225)** | 1.065 (0.997; 1.139) | **0.795 (0.742; 0.852)** | **1.510 (1.383; 1.650)** |
| Hydromorphone | 1.015 (0.921; 1.120) | **1.105 (1.000; 1.221)** | 1.087 (0.985; 1.199) | 1.082 (0.937; 1.248) |
| Comorbidities |  |  |  |  |
| Chronic infections | **0.721 (0.537; 0.970)** | **10.644 (7.346; 15.424)** | 1.195 (0.906; 1.576) | 0.756 (0.437; 1.307) |
| Chronic inflammatory disease | **1.498 (1.440; 1.558)** | **1.253 (1.206; 1.301)** | **1.366 (1.314; 1.421)** | **0.819 (0.771; 0.870)** |
| Renal disease | 1.023 (0.860; 1.216) | **1.289 (1.071; 1.551)** | **1.312 (1.107; 1.555)** | **1.615 (1.291; 2.021)** |
| End stage renal disease | 1.013 (0.828; 1.239) | **1.580 (1.269; 1.966)** | **2.149 (1.762; 2.622)** | **1.356 (1.051; 1.751)** |
| Diabetes | 1.036 (0.981; 1.095) | **1.281 (1.213; 1.353)** | **1.204 (1.140; 1.270)** | 1.012 (0.934; 1.097) |
| Pulmonary disease | **1.294 (1.231; 1.360)** | **2.076 (1.968; 2.191)** | **1.283 (1.220; 1.349)** | **1.256 (1.167; 1.353)** |
| Liver failure | **1.689 (1.254; 2.274)** | **0.122 (0.084; 0.177)** | 0.797 (0.603; 1.054) | 1.613 (0.932; 2.789) |
| Organ transplant | 1.044 (0.762; 1.432) | **0.258 (0.177; 0.376)** | 1.153 (0.855; 1.556) | 1.230 (0.702; 2.155) |
| Neurologic disease | 1.066 (0.991; 1.146) | **1.175 (1.088; 1.269)** | **1.139 (1.058; 1.226)** | **1.120 (1.017; 1.234)** |
| Cardiac disease | **1.168 (1.117; 1.221)** | **1.512 (1.451; 1.575)** | **1.871 (1.788; 1.958)** | 0.793 (0.745; 0.844) |
| Thyroid disease | 1.041 (0.975; 1.112) | **1.126 (1.054; 1.204)** | **1.173 (1.099; 1.251)** | 0.909 (0.824; 1.002) |
| Gout | **1.187 (1.087; 1.295)** | **1.134 (1.034; 1.244)** | **1.152 (1.056; 1.258)** | 0.900 (0.791; 1.025) |
| Psychiatric disease | **1.191 (1.144; 1.240)** | **1.325 (1.275; 1.377)** | **1.132 (1.087; 1.179)** | **1.451 (1.371; 1.537)** |
