# Supplementary material for: Increased risk of adverse events in non-cancer patients with chronic and high-dose opioid use—A health insurance claims analysis
Source: PLoS One. 2020 Sep 14;15(9):e0238285. doi: 10.1371/journal.pone.0238285 (PMC7489518; doi:10.1371/journal.pone.0238285)
Supplement: Attachment — Submitted filename: PONE-D-19-34609-Response to Reviewers-17.02.2020.docx [file pone.0238285.s005.docx]

Response to Reviewers – PONE-D-19-34609

Dear Vijayaprakash Suppiah, Academic Editor,

Highly esteemed Reviewers:

Thank you for considering our manuscript entitled *“Increased risk of adverse events in non-cancer patients with chronic and high-dose opioid use – a health insurance claims analysis”* for publication in PlosOne and for giving us the opportunity to submit a revised version.

We thank the reviewers and the editor for their thorough review and the very important comments. We changed our manuscript accordingly and we believe that the revised manuscript improved substantially. We address the reviewers’ comments in detail below.

**Responses to Reviewer's Questions**

**Reviewer #1:**

Comment #1: This is an interesting and important paper but with one challenging statistical design issue. It appeared that longer duration (e.g., more than 360 days of opioid use) generally leads to lower risk of death, but one needs to be alive to complete 360 days of opioid use -- thus there is most likely a mechanical association between opioid use duration and mortality.

On page 13, line 4: "The majority of patients, died during an acute episode" I would write it differently, that the majority of deaths occurred during an acute episode.

**Answer:** We agree that adjusting for the episode duration may suggest that the longer you use opioids, the less likely you are to die. It is important to note, that we do not have indications for the opioid use. The higher mortality rate earlier is most likely related to the indication of the opioid and in this case, the treatment may be also relieving symptoms at the end of life.

We reframed the results section to address this point (p. 12, line 24):

“The majority of deaths occurred during an acute episode (n=4,309, 44.6%). Deaths occurred in 5.8% during subacute (n=562), in 22.5% during chronic (n=2,173), and in 27.1% in very chronic (n=2,618).”

Comment #2: Finally, I would have liked to see more results using actual percentages related to adverse outcomes, rather than odds ratios, for two reasons. First, knowing the likelihood of adverse events is always helpful to get a context of how important the problem is, and second, odds ratios have well-known interpretation issues when the likelihood of the adverse event is high. It may not be much of a problem here, but I would like to see something there.

**Answer:** the reviewer addresses an important point. We included the number and percentages for the outcomes in a results table (new Table 2).

We also added the information in Table 2 in the specific sections of the manuscript:

- ED visits: p. 13, line 5
- Infection: p. 13, line 16
- Hospitalization: p. 14, line 2
- Death: p. 14, line 11

**Reviewer #2:**

In this study a large Swiss insurance database was used to estimate adverse events associated with different levels and durations of opioid prescribing. The outcomes are largely in line with those of previous studies although the Swiss population perhaps has not been the focus of previous analyses.

1. The WHO ladder was not intended nor validated to be used for non-cancer pain. Certainly, there has been a massive increase in opioid prescribing in many countries, but it is not clear that mistaken adherence to a now very old cancer-related algorithm is much to blame. Aren’t there much stronger reasons like pharma company promotion, efforts of advocacy groups and strongly expressed although poorly justified opinions of “experts?” Perhaps these should be included in the first paragraph.

**Answer:** we agree with the reviewers comment that many other factors were involved in driving the opioid prescriptions. We expanded the introduction as follows (p 4, line 9)

“Advocacy for pain control, advertisement of efficacy of opioids for chronic pain based on low level evidence, and aggressive prescription practices by physicians resulted in an increased use of opioids for cancer and non-cancer related pain (4-6).”

2. The description of medical benefits is appreciated. However, some clear statement of the lowlihood of capturing most or all opioid prescriptions for the cohort in the database would be helpful.

**Answer:** Thank you for raising this point. We added the information (p 6, line 4):

“The Swiss health care system is highly decentralized and no centralized opioid registry is available. Opioids cannot be purchased over the counter and for strong opioids, a special prescription with a unique identification number (a so called “prescription for narcotic substances”) is issued with 3 copies. One copy remains with the prescribing physician, one with the pharmacy, and one with the insurance company. Although the regulation minimizes the risk of abuse, potential misuse (e.g. multiple prescribers) cannot be identified because no central database exists. Further, pharmacies send their bills directly to the insurance company. Therefore, opioid claims cover close to 100% of all prescribed opioids.”

3. Episodes occurring within the first week after prescribing were excluded because they were thought to be likely related directly to the opioid. These may in fact be some of the most interesting occurrences! It would be OK to analyze these separately, but please do include them along with prescribed dose relationships. Also, it was confusing why a 6-day period of exclusion for outcome was described in one section of the methods and a 2 week period seemed to be described in the “Outcome of interest” section.

**Answer:** Thank you for detecting this mistake. We removed from the “Medication Exposure” section the sentence “We did not include outcomes that occurred within the first 6 days because they were most likely directly related to the opioid prescription.” (p. 7, line 19).

We included all opioid episodes. We considered also all outcomes. But as the study aimed to assess the risk of adverse events (events associated with opioid use itself and not with why opioids were prescribed), we did not include outcomes that occurred within the first 2 weeks in the statistical models.

We specified the outcome of interest section as follows (p 9, line 20):

“According to the definition of the WHO, adverse events are medical occurances temporally associated with the use of a medical product, but not necessarily causally related. Adverse events were included in the analysis when they occurred two weeks or later after the index date of the opioid prescription.”

4. Is there validation or even rationale for using the identity of the pharmacological agent as a proxy for the complexity of the episode? Why does this matter and couldn’t choice be dues to the arbitrary practices of individual providers?

**Answer:** While we agree that we cannot fully exclude arbitrary practices of individual providers, we see in clinical practices preferences for specific pain conditions (e.g. in Switzerland Oxycodon / Naloxon combinations are frequently used for musculoskeletal pain, fentanyl patches in elderly women, and morphine at the end of life). We also agree with the reviewer, that the interpretation of the Odds ratios from the model may lead to misinterpretation on the specific safety of a pharmacological agent. We therefore, removed them from the tables and the figures and now added the information that the models were adjusted for the pharmacological agents (Table and Figure legends).

While the specific choice may be influenced by the physician’s preferences, this does not relate to the changes from one agent to the other. Changing of pharmacological agents within an episode indicates poor pain control. As we do not have information on the indication, we used the pharmaceutical agent in our model as a proxy for episode complexity. We believe that adjusting for pharmacological agents in our model is a distinct strength of our study, as it allows us to adjust for the missing of information on indication and pain control.

We expanded the methods section as follows (p 10, line 23): “Additionally, we included the pharmacological agent of the strong opioid (i.e. morphine, oxycodone, buprenorphine, fentanyl, hydromorphone, and pethidine) as a proxy for the complexity of an episode (e.g. opioid rotation with changes of substances during an episode or preferred use of a substance depending on the clinical situation). As we did not have information on the indication for opioid prescription and pain control, pharmacological agents within one episode may indicate complexity of pain problems.”

5. Please clarify whether opioid overdose cold have been included as an adverse effect, or even a diagnosis related to substance misuse. These would be much easier to interpret.

**Answer:** We agree that both – opioid overdose and substance misuse – would have been easier to interpret. As we did not have diagnostic codes available in the insurance claims database, we were not able to include them into the analysis. Further, we excluded all patients that fulfilled our drug substitution criteria for opioid use disorders. As Switzerland has a drug substitution program since the 1990ies that is also reimbursed by insurance companies, it is quite difficult to distinguish between misuse and opioid dependency.

We added the information to the methods section (p 10, line 5): “Because no information on diagnoses and misuse were available in the insurance claims database, we were not able to assess these two outcomes.”

6. Acute opioid prescribing seems to have been used as the reference. Why not a propensity-matched no-opioid group? Unless there is a very compelling reasons not to do this, a no-opioid matched group should be included.

**Answer:** Thank you for this comment, please let us clarify what we did. We used the group of acute opioid episodes as reference category in the multiple logistic regression models, to estimate the contrasts of subacute, chronic, and very chronic episodes for different outcomes. In our analysis, the unit of analysis was opioid episode, with different characteristics and duration. We agree with the reviewer in principle that propensity score matching could be used for the analysis of observational data. However, in this study, the opioid episodes would have no corresponding non-opioid episodes in the database, which would make (propensity score) matching infeasible.

7. Tables are very hard to read by themselves. Please make better use of graphical representations of key data.

**Answer:** Based on reviewer 1 and your comments we included a results table (Table 2) for the main outcome of interest. This will allow the reader to assess the absolute unadjusted differences between opioid dose groups and treatment duration.

We also simplified Table 3, Figure 1 and 2.

**Editorial comments**

1. Please ensure that your manuscript meets PLOS ONE's style requirements, including those for file naming. The PLOS ONE style templates can be found at

<http://www.journals.plos.org/plosone/s/file?id=wjVg/PLOSOne_formatting_sample_main_body.pdf> and <http://www.journals.plos.org/plosone/s/file?id=ba62/PLOSOne_formatting_sample_title_authors_affiliations.pdf>

**Answer:** the style of the manuscript was changed according to the PlosOne style requirements.

2. Please correct your reference to "p=0.000" to "p<0.001" or as similarly appropriate, as p values cannot equal zero.

Answer: the manuscript should not contain p=0.000. In Table 1 p values are <0.000.

3. We note that you have indicated that data from this study are available upon request. PLOS only allows data to be available upon request if there are legal or ethical restrictions on sharing data publicly. For information on unacceptable data access restrictions, please see <http://journals.plos.org/plosone/s/data-availability#loc-unacceptable-data-access-restrictions>.

In your revised cover letter, please address the following prompts:

a) If there are ethical or legal restrictions on sharing a de-identified data set, please explain them in detail (e.g., data contain potentially identifying or sensitive patient information) and who has imposed them (e.g., an ethics committee). Please also provide contact information for a data access committee, ethics committee, or other institutional body to which data requests may be sent.

b) If there are no restrictions, please upload the minimal anonymized data set necessary to replicate your study findings as either Supporting Information files or to a stable, public repository and provide us with the relevant URLs, DOIs, or accession numbers. Please see <http://www.bmj.com/content/340/bmj.c181.long> for guidelines on how to de-identify and prepare clinical data for publication. For a list of acceptable repositories, please see <http://journals.plos.org/plosone/s/data-availability#loc-recommended-repositories>.

We will update your Data Availability statement on your behalf to reflect the information you provide.

**Answer:** We updated the data availability statement as follows:

This study is based on administrative, de-identified insurance claims data handled in compliance with privacy law and regulations. The analyses were performed within the premises of the Department of Health Sciences at the Helsana and only aggregated data was shared. The study was conducted following the principles of good clinical practice and in accordance with the Declaration of Helsinki. The Helsana Group approved the study protocol. Aggregated data can be made available only on reasonable request after assessment and permission of the data owner because the data is part of the claims database of the Helsana Insurances Group. Thus, data underlie data strict protection and privacy restrictions. These restrictions prohibit us from sharing the collected data.

Deidentified dataset including the variables used for the analysis can be made available upon request from the Helsana Group. Please contact:

Department of Health Sciences, Helsana,

Zürichstrasse 130

CH-8600 Dübendorf

Switzerland

https://www.helsana.ch/de/private

Phone: +41 58 340 18 80

Gesundheitskompetenz@helsana.ch

The authors confirm that they had access to the data required to perform the analysis. The authors conform that they did not have any special access to this data which other researchers would not have.

The authors confirm that they had access to the data required to perform the analysis.

2. Provide a website, email address, or phone number for researchers to contact the Helsana Group. According to the local ethical committee (Ethical committee of the Canton Zurich, Switzerland) no IRB approval was required.

4. Thank you for stating the following in the Competing Interests section:

"MW, JB, JS, and UH have no competing interest to declare. AS and EB are employed by the Helsana Group. The sponsor had no role in the planning, conducting or submission of this manuscript. These authors declare no conflict of interest. Helsana Group shall have no liability to any third party in respect to the contents of this article. All the other authors have no conflict of interests or financial disclosures to declare."

Please confirm that this does not alter your adherence to all PLOS ONE policies on sharing data and materials, by including the following statement: "This does not alter our adherence to  PLOS ONE policies on sharing data and materials.” (as detailed online in our guide for authors <http://journals.plos.org/plosone/s/competing-interests>).  If there are restrictions on sharing of data and/or materials, please state these. Please note that we cannot proceed with consideration of your article until this information has been declared.

Please include your updated Competing Interests statement in your cover letter; we will change the online submission form on your behalf.

**Answer:** the competing interest statement is included in the cover letter.

5. Your ethics statement must appear in the Methods section of your manuscript. If your ethics statement is written in any section besides the Methods, please move it to the Methods section and delete it from any other section. Please also ensure that your ethics statement is included in your manuscript, as the ethics section of your online submission will not be published alongside your manuscript.

**Answer:** the ethics statement is no included in the methods section.

6. Please include your tables as part of your main manuscript and remove the individual files. ** Please note that supplementary tables (should remain/ be uploaded) as separate "supporting information" files **

**Answer:** the tables are now included in the main manuscript.
