# Supplementary material for: Increased risk of adverse events in non-cancer patients with chronic and high-dose opioid use—A health insurance claims analysis
Source: PLoS One. 2020 Sep 14;15(9):e0238285. doi: 10.1371/journal.pone.0238285 (PMC7489518; doi:10.1371/journal.pone.0238285)
Supplement: Attachment — Submitted filename: PONE-D-19-34609-Response to Reviewers-r2-04.05.2020.docx [file pone.0238285.s006.docx]

Response to Reviewers – PONE-D-19-34609

Dear Vijayaprakash Suppiah, Academic Editor,

Highly esteemed Reviewers:

Thank you for considering our manuscript entitled *“Increased risk of adverse events in non-cancer patients with chronic and high-dose opioid use – a health insurance claims analysis”* for publication in PlosOne and for giving us the opportunity to submit a revised version.

We thank the reviewers and the editor for their thorough review and the very important comments. We changed our manuscript accordingly and we believe that the revised manuscript improved substantially. We address the reviewers’ comments in detail below.

**Responses to Reviewer's Questions**

**Reviewer #4:**

**Comment #1:** You exclude cancer-related opioid treatment. Are there other conditions that you found were treated with opioids at a high rate?

**Answer:** We now include a description of the most prevalent non-cancer diseases in the revised Results section (page 12, lines 11-17). In the study population, the most prevalent treatments observed were for cardiac diseases (60.8%), psychiatric conditions (42.2%), chronic inflammatory diseases (37.7), pulmonary diseases (14.3%), for chronic infections (10.5%), and diabetes (12.1%). Compared to the acute opioid episodes, the proportions increased in very chronic episodes for cardiac diseases (40.3 to 81.2%), psychiatric diseases (21.4 to 66.9%), inflammatory diseases (21.3 to 81.2%), pulmonary diseases (6.6 to 24.7%), chronic infections (5.3 to 18.5%), and diabetes (7.0 to 17.2%).

**Comment #2:** Are there combinations of medical conditions that also lead to high opioid use and treatment? That combination of conditions could also contribute to adverse medical outcomes. Correspondingly, a recommendation could be that for a patient with this set of conditions faces more adverse medical outcomes from strong opioid treatment.

**Answer:** The reviewer raises an important point. To address the reviewers’ comment, we checked for significant interactions in our models. We found almost no effect on the model fit. The effect sizes for opioid treatment duration, opioid maximum dose, and benzodiazepines were robust. When interactions were retained in backward stepwise procedures using Akaike information criterion (AIC), lung diseases were often involved but then again removed throughout the backward elimination process using the Bayesian information criterion (BIC). Based on the missing impact of the interactions, we decided to keep the simple model containing only the main effects.

In our prediction models chronic inflammatory diseases, pulmonary diseases, and psychiatric diseases were consistently associated with an increased risk for all outcomes. For other comorbidities, the associations were less clear. We found an increased risk associated with the treatment for renal and neurologic disease for infections, hospitalizations, and death but not for ED visits. Treatments for cardiac diseases and gout were associated with an increased risk for ED visits, infections, and hospitalization but not death. To demonstrate these associations, we now added a supplementary file including the full models (**S4 Table**). We also include a section on the influence of comorbidities in the revised results section (page 14, lines 15-22). As the association observed in our models cannot infer causality, we also discuss this in the revised Discussion (page 16, lines 10-15) and Limitations section (page 16, lines 23-25) and highlight the need for additional studies to investigate the potential causal factors (page 17, lines 10-14).
